# Polyphenols, Flavonoids, and Antioxidant Activity Involved in Salt Tolerance in Wheat, *Aegilops cylindrica* and Their Amphidiploids

**DOI:** 10.3389/fpls.2021.646221

**Published:** 2021-03-25

**Authors:** Razieh Kiani, Ahmad Arzani, S. A. M. Mirmohammady Maibody

**Affiliations:** Department of Agronomy and Plant Breeding, College of Agriculture, Isfahan University of Technology, Isfahan, Iran

**Keywords:** antioxidant, biochemical traits, phenol, ROS, salinity stress

## Abstract

*Aegilops* spp. is the closest genus to wheat (*Triticum* spp.), which makes *Aegilops* great candidates to exhibit precursors of wheat features. *Aegilops cylindrica* Host displays excellent salt tolerance. In the current study, biochemical and phytochemical compounds in the leaves of two wheat cultivars, one hyper-salt tolerant *Ae. cylindrica* genotype and their amphidiploids (derived from “Chinese Spring” × *Ae. cilindrica* and “Roshan” × *Ae. cylindrica*), grown under control and saline field conditions, were assessed. These compounds included total protein content, proline content, electrolyte leakage, total flavonoid content, total phenolic content, DPPH radical scavenging activity, and reducing power. In addition, phenolic components were also identified using HPLC analysis. Chlorogenic acid, ellagic acid, ferulic acid, syringic acid, vanillic acid, p-coumaric acid, caffeic acid, and gallic acid were the most abundant phenolic acids. Luteolin, apigenin, and rutin were the most abundant flavonoids in the leaves. Salt stress significantly increased all biochemical variables, with the exceptions of reducing power and p-coumaric acid. Interestingly, amphidiploid genotypes exhibited intermediate levels of most of the detected phenolic compounds between the two parental species. As demonstrated by bivariate correlations luteolin, chlorogenic acid, caffeic acid and apigenin could predict inhibition percentage by DPPH assay, suggesting a possible role in the cellular defense against oxidative stress in wheat. The amphidiploids and their wild parent performed significantly better than wheat cultivars on phenolic constituents, flavonoids, and maintaining redox homeostasis under salt stress conditions.

## Introduction

Wild relatives of wheat are extremely valuable genetic resources for the continued improvement of bread wheat, the most important and widely grown cereal that, as a staple food, contributes significantly to the diet of the world population (Arzani and Ashraf, 2017). Due to the demands and challenges of the future, such as world population growth, erosion of genetic resources, and global climate changes, diverse gene pools are needed to broaden the genetic base of modern crop cultivars (Arzani and Ashraf, 2016). *Aegilops cylindrica* Host is a wild species, sharing a common genome (D) with common wheat (*Triticum aestivum* L.). Several authors (e.g., Kimber and Sears, 1987; Goncharov, 2011) recommended that the *Aegilops* species be classified in the *Triticum* genus, because of their close relationship.

Salinity is a significant abiotic stress that severely limits crop growth and productivity worldwide. Salinity causes osmotic stress, ion toxicity, nutritional disorders, and oxidative stress (Munns and Tester, 2008; Arzani and Ashraf, 2016). These factors can cause physiological and biochemical defects in plant tissues. Plant cells generate various phenolic compounds as the low molecular weight non-enzymatic antioxidants which aid in removing the reactive oxygen species (ROS) (Hodaei et al., 2018). Phenolic compounds have shown to be effective to protect biological systems against various oxidative stresses, playing crucial role in maintaining redox-homeostasis, and offering a potential targets for improving stress tolerance in plants (Trchounian et al., 2016). Therefore, plant adaptation to salt stress is one of the possibility affected by the homeostasis between ROS and phytochemicals such as polyphenols and flavonoids.

Phenolic compounds, including flavonoids, are the most widely distributed secondary metabolites present in the plant kingdom. These compounds play numerous biochemical and molecular roles in the plants, such as signaling molecules, plant defense, mediating auxin transport, antioxidant activity, and free radical scavenging (Tohidi et al., 2017). Among non-enzymatic antioxidants, phenols and flavonoids contribute significantly as scavenging free radicals in the plants for tolerating salt stress by accumulating in various tissues (Sirin and Aslım, 2019). Polyphenols are present in free and bound forms in plant materials. Phenolic acids represent the central portion of polyphenols present in the grains and baked products in cereal, and around 75% of these are available in the bound form (Ceccaroni et al., 2020).

The synthesis and accumulation of phytochemical compositions in plant tissues are influenced by the genotype, growing environment, and their interaction. Phenolic compounds and flavonoids of the grains were assessed in wheat genotypes under normal conditions (Dinelli et al., 2011; Shewry and Hey, 2015), temperature stress (Ceccaroni et al., 2020), and salt stress (Stagnari et al., 2017) during germination. The quantity and composition of polyphenols of the grain, flour, and baked-products are essential for human health. Still, knowledge about the green, photosynthesizing assimilating tissues (source) from where the substrate materials and metabolites transfer to storage organs (grains) is limited. However, limited research has addressed the response of wheat and *Ae. cylindrica* genotypes in terms of phytochemicals accumulation in the leaves of plants grown under salt stress conditions.

The purpose of this study was to determine how salt stress affects the leaf polyphenol composition, flavonoids, and cellular redox responses to oxidative stress in two wheat cultivars (including “Chinese Spring” and “Roshan”), one *Aegilops cylindrica* genotype and their two amphidiploids (F_1_ hybrids) derived from cross between “Chinese Spring” and “Roshan” wheat cultivar with *Ae. cylindrica* as the male parent.

## Materials and Methods

### Plant Materials

Five genotypes, including two wheat cultivars (“Chinese Spring” and “Roshan”), one *Ae. cylindrica* genotype (no. 56), and two of their interspecific hybrids (amphidiploids) were used in this study. Two common wheat (*Triticum aestivum* L.) cultivars (“Chinese Spring” and “Roshan”) as the female parents were crossed with a highly salt-tolerant genotype (Kiani et al., 2015) of *Aegilops cylindrica* as the male parent. Fourteen days after pollination, the immature embryos were cultured in the Murashige and Skoog (MS). Subsequently, the colchicine treatment was carried out at the 4–5 tiller stage and the treated plants were kept under greenhouse conditions until they were harvested. Full details of the hybridization protocols, including emasculation, pollination, growth hormone treatment, embryo rescue, and colchicine treatment of amphihaploid plants, have been described in an unpublished article.

### Experimental Conditions

The seeds of amphidiploid genotypes and their parents were planted in the research farm of the Isfahan University of Technology, Lavark, Iran (40 km south-west of Isfahan; 32° 32′ N, 51° 23′ E; 1630 m asl) in 2017-2018 growing season. The soil in the top 60-cm layer is a clay loam (pH 7.5) with an electrical conductivity (EC) of 2.4 dS m^–1^. For each of two experiments [control and saline (250 mM NaCl)], a randomized complete block design replicated three times was used. Each plot was three rows, 1 m long, with a row spacing of 30 cm. In the control experiment, plants were irrigated with freshwater (EC = 1 dS m^–1^). In the saline experiment, fresh water was applied until the four tillers stage (Zadok’s scale 24) but later shifted to saline water. The experimental plots received equal irrigation, monitored by flow meters, in the control and saline conditions. The saline experiment was irrigated with irrigation water connected to the upstream tank that delivered a concentrated solution of sodium chloride (1 M NaCl) to maintain the desired concentration of salinity (250 mM NaCl) using a calibrated flow meter. The plots were irrigated when the soil moisture reached above 80% of field capacity (ψ = –0.06) in the root zone. The soil ECe, in 0 – 40 cm soil depth, was measured in all plots at harvesting stages. The average soil ECe values were 2.4 and 15.4 dS m^–1^ for the control and saline field conditions, respectively.

### Biochemical Properties

#### Total Protein Content

Total protein content of leaf samples was determined at the grain filling stage according to Bradford (1976) using bovine serum albumin as a standard. Total protein content was expressed as milligram per gram leaf dry weight (mg g DW^–1^).

#### Proline Content

Free proline was extracted from the fresh leaves, derivatized with acid ninhydrin and absorbance read, according to Bates et al. (1973) method. Fresh leaf (0.5 g) collected from both control and salinity stressed plants, quickly frozen and ground in liquid nitrogen. The sample was homogenized in 10 ml sulfosalicylic acid (3% w v^–1^), and centrifuged for 10 min at 8,500 rpm. Then, 2 ml of the supernatant was added to a mix soluble of 2 ml ninhydrin reagent and 2 ml acetic acid and kept in a water bath (100 °C) for 1 h. Subsequently, 4.0 mL of toluene was added to the samples and absorbance was recorded at 520 nm. Proline concentration was calculated using a standard curve according to the following formula:

$$\text{Proline}\left(\mu \text{M}/{\text{g}}^{-1}\text{FW}\right)=\frac{\left(\mu g\mathrm{Proline}/\mathrm{mL}\times \mathrm{mL}\mathrm{Toluene}\right)\times 5}{\left(115.5\mu g/\mu \text{M}\times g\mathrm{sample}\right)}$$

#### Total Phenolic Content (TPC)

First, 1.25 g of the dried leaf samples was mixed with 25 mL of 80% methanol in an orbital shaker (150 rpm) at 25°C for 24 h. Total phenolic content (TPC) of the methanolic extract solution was determined using Folin–Ciocalteu method as described by Tohidi et al. (2017) with minor modification. Briefly, 0.5 ml of the filtered methanolic extract was added to a mixture of 2.5 ml of the Folin–Ciocalteu reagent (diluted 10-fold) and 2 ml of 7.5% sodium carbonate in a test tube and shaken well. After 15 min heating at 45°C, the absorbance was read at 765 nm using a spectrophotometer against a blank solution. Tannic acid was used as a standard for total phenol quantification. Thus, the data have been expressed as mg tannic acid equivalent (TAE) per g DW.

#### Total Flavonoid Content (TFC)

The aluminum chloride assay was used to determine total flavonoid content of the methanolic extract solution (Tohidi et al., 2017). An aliquot of 125 μl of the extract solution was added to 75 μl of 5% NaNO_2_ solution. The mixture was then allowed to stand for 5 min before adding 150 μl of aluminum chloride (10%) solution. After that, 750 μl of NaOH solution (1 M) was added and the final volume of the mixture was adjusted to 2500 μl by deionized water. After an incubation period of 15 min, the absorbance was read at 510 nm using a spectrophotometer. The TFC has been expressed as mg quercetin equivalent (QE) per g dry weight (DW).

#### DPPH Radical Scavenging Activity

The DPPH radical scavenging activity of the leaf samples was determined by the procedure described by Tohidi et al. (2017). Briefly, 0.1 ml of the sample of each plant extract was blended at chosen concentrations (50, 100, and 300 ppm) in which the initial and final absorbance values of the initial amount of DPPH in BHT standard was within the range of accuracy of spectrophotometry (Sharma and Bhat, 2009). The sample volume, 5 mL of 0.1 mM methanol DPPH solution selected was appropriate for obtaining good results. The mixture was then shaken vigorously and incubated for 30 min in the dark at room temperature. The absorbance of the mixture was read at 517 nm (AA) and corrected for the absorbance of blank reagent (AB) at the same wavelength. A DPPH-methanol solution containing 80% methanol was used as a negative control (AB). The synthetic antioxidant reagent, butylated hydroxytoluene (BHT), was used as a positive control. The IC50 value (μg ml^–1)^, the concentration in μM that inhibits DPPH absorption by 50%, was calculated by linear regression analysis.

#### Reducing Power Assay

The reducing power of the leaf samples was determined according to the method described by Tohidi et al. (2017), with slight modifications. In 2.5 ml of leaf extract (at concentrations of 0.5, 1, 3, and 5 mg ml^–1^) was added to 2.5 ml of 1% potassium ferricyanide and 2.5 ml 0.2 M phosphate buffer (pH = 6.6). The mixture was incubated at 50°C for 20 min, and 2.5 ml of trichloroacetic acid (10%) was then added. The homogenate was centrifuged at 3000 rpm for 10 min, and 2.5 ml of the supernatant was mixed with 2.5 ml of ferric chloride (0.5 ml, 0.1%) and deionized water. The absorbance of the solution was read at 532 nm against a blank. The antiradical activity was expressed as IC50 (mg ml^–1^). The IC50 was calculated using a linear regression equation.

#### Polyphenol Composition

Individual phenolic acids of the leaves were analyzed using the high-performance liquid chromatography (HPLC) system. An HP 1090 series HPLC (Agilent Technologies, United States) equipped with a Waters Symmetry^®^ C18 column (4.6 × 250 mm, particle size 5 μM) and a UV absorbance detector was used. The extracts were filtered using a 0.45 μM nylon membrane filter (Whatman Inc., Maidstone, United Kingdom), and 20 μL was injected into the analytical column. Eluent A was 0.1% formic acid in water (v/v), and eluent B was 0.1% formic acid in acetonitrile (v/v). The flow rate was 0.8 mL min^–1^, and the following linear gradient program was used: 10% to 26% solvent B for 20 min, 65% solvent B for 40 min, and finally to 100% solvent B for 45 min. Detection was performed at 200–400 nm using the absorbance detector. Individual phenolic acids, flavonoids, and flavanols in the leaf samples were determined and quantified by the assessment of their relative retention times to the authentic standards. The data have been expressed as milligrams phenolic acid per 100 g of DW (mg 100 g^–1^ DW).

#### Electrolyte Leakage

Electrolyte leakage (EL) was used to assess membrane permeability. The EL was determined at the grain filling stage using an electrical conductivity (EC) meter as described by Lutts et al. (1996). The EL (%) was calculated using EL (%) = [1 – (EC1/EC2)] × 100 where, EC1 and EC2 are the electrical conductivities of the fresh leaf bathing solution before and after incubation in boiling water, respectively.

#### Grain Yield

At maturity, ten plants from the middle of each plot were harvested. Grain yield was recorded per plant (*n* = 10) and expressed as g per plant.

#### Statistical Analysis

The data were analyzed by analysis of variance (ANOVA) using the PROC GLM of SAS (version 9.4; SAS Institute Inc., Cary, NC, United States). The means were further analyzed by Fisher’s protected least significant difference (LSD) test at *p* < 0.05. The PROC CORR of SAS was used to calculate the correlation coefficients. A backward stepwise regression analysis was also carried out using grain yield as the dependent variable and protein content, proline content, El, TPC, TFC, and reducing power as the independent variables by SPSS software version 18.0 (SPSS Inc., Chicago, IL, United States).

## Results

Statistical evaluation of the effects of salt stress, genotype, and salt stress × genotype interaction on biochemical traits was carried out, and the results of ANOVA are given in Table 1. Salt stress resulted in significant changes (*p* < 0.01) to the biochemical traits in the leaf. The genotypes were significantly different for these traits. Also, significant genotype × salt stress interaction was found for all biochemical traits except for total protein content (Table 1).

**TABLE 1:** Results of analysis of variance for the biochemical properties and grain yield of the genotypes studied under the control and salt stress conditions.

	df	Protein content	Proline content	EL^♣^	DPPH	Reducing power	TPC	TFC	Grain yield
Replication	2	0.001	1.01	0.13	103	0.001	0.01	0.001	127.3
Salt stress (S)	1	0.611**	499.08**	2283.3**	411738144.0**	6283763.3**	39.46^ **^	22.80**	12934.8**
Genotype (G)	4	0.15**	52.64**	210.59**	31088627.0**	40933566.7**	92.39^ **^	3.03**	6223.7**
G × S	4	0.01^*ns*^	45.58*	181.47*	6763986.0**	11597276.3**	8.363^ *^	2.89**	2035.7**
Residual	18	0.02	1.23	15.89	18	1.9	0.83	0.003	242.5
CV (%)		9.48	12.52	7.85	0.62	3.03	6.68	1.11	26.42

^ns^ not significant. * and ** significant at the p < 0.05 and p < 0.01, respectively. ^♣^EL, electrolyte leakage; DPPH, 2, 2-diphenyl-1-picryl-hydrazyl-hydrate; TPC, total phenolic contents; TFC, total flavonoid content.

### Total Protein Content

**FIGURE 1:**
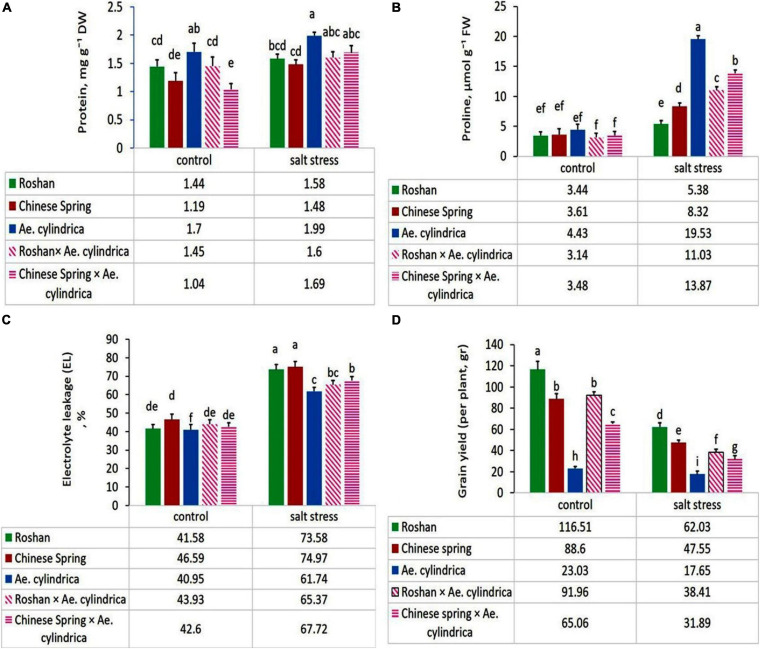
Mean comparison of **(A)** total protein content, **(B)** proline content, **(C)** electrolyte leakage, and **(D)** grain yield of parents (*T. aestivum* “Roshan,” “Chinese spring,” *Ae. cylindrica*) and their F_1_ hybrid plants (amphidiploids) under control and salt stress conditions. Bars represent means ± SE and bars with the same letter do not significantly differ at *P* < 0.05 among the genotypes.

Significant increase in the total protein content in the leaves of plants was found in salt stress conditions compared with control one (Table 2). Mean comparison of the genotypes showed that the genotype of *Ae. cylindrica* had the highest total protein content under salt stress and control (Figure 1A).

**TABLE 2:** Means (±SE) of biochemical properties and grain yield of the genotypes studied under control and salt stress conditions.

	Control (0 mM NaCl)	Salt stress (250 mM NaCl)	LSD (*P* < 0.05)	Change (%)
**Biochemical traits**				
Protein content (mg g DW^–1^)	1.43 ± 0.02	1.71 ± 0.02	0.11	19.58
Proline content (μmol g FW^–1^)	3.87 ± 0.08	7.02 ± 0.54	0.76	37.42
Electrolyte leakage (%)	48.63 ± 0.71	66.07 ± 0.94	3.09	35.86
TPC^♣^ (mg TAE g^–1^ DW),	12.88 ± 0.43	15.18 ± 0.34	0.71	17.86
TFC (mg QE g^–1^ DW)	3.81 ± 0.08	5.56 ± 0.10	0.04	45.93
DPPH (IC50, μg ml^–1^)	1329.80 ± 91.57	1806.4 ± 34.29	3.32	36.84
Reducing power (IC50, mg ml^–1^)	1.31 ± 0.04	0.83 ± 0.01	1.08	−36.64
Grain yield (g plant^–1^)	79.70 ± 3.29	38.17 ± 1.26	12.05	−52.11

*^♣^TPC, total phenolic contents; TFC, total flavonoid content; DPPH, 2, 2-diphenyl-1-picryl-hydrazyl-hydrate.*

### Proline Content

The results of ANOVA showed that leaf proline content was significantly affected by salt stress, genotype, and their interaction (Table 1). Exposure to 250 mM NaCl resulted in an overall twofold increase in proline content (Table 2). *Ae. cylindrica* genotype had the highest proline content in both control and salt stress conditions (4.43 and 19.53 μM g^–1^ FW, respectively), which are much higher than those found for the “Chinese Spring” and “Roshan” wheat cultivars. Interestingly, the interspecific hybrid (amphidiploid) plants displayed an intermediate phenotype between male (*Ae*. *cylindrica*) and female (wheat) parents (Figure 1B).

### Electrolyte Leakage (EL)

The results of the current study showed a significant increase in leaf EL in response to salt stress, as expected (Table 2). However, a significant genotypic difference was observed for the EL response under salt stress, with the highest values seen in wheat cultivars “Chinese Spring” (74.97%) and “Roshan” (73.58%) and *Ae. cylindrica* the lowest (61.74%) (Figure 1C).

### Grain Yield

Grain yield was affected significantly by salt stress, genotype, and salt stress × genotype (Table 1). Salt stress significantly decreased in all study genotypes due to salt stress (Figure 1D). Yield loss, the outcome of the physiological and biochemical changes, was overall found to be 52% (Table 2). Wheat by *Ae. cylindrica* derived amphidiploids showed less yield loss than wheat cultivars (“Roshan” and “Chinese Spring”). In contrast, *Ae. cylindrica* genotype produced the least loss of yield (data not shown).

### Total Phenolic and Flavonoid Content

**FIGURE 2:**
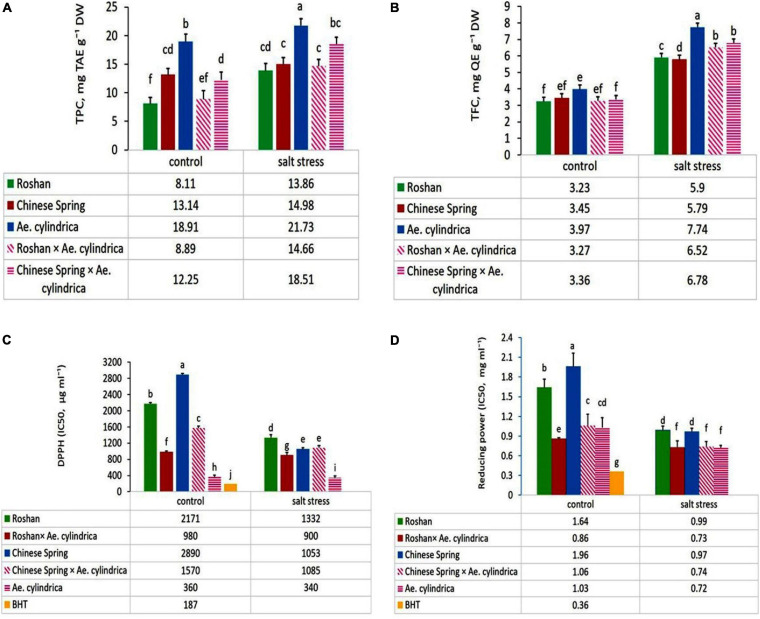
Mean comparison of **(A)** total phenolic contents (TPC, mg TAE g^–1^ DW), **(B)** total flavonoid content (TFC, mg QE g^–1^ DW), **(C)** DPPH (IC50, μg ml^–1^), and **(D)** reducing power (IC50, mg ml^–1^) of parents (*T. aestivum* “Roshan,” “Chinese spring,” *Ae. cylindrica*) and their F_1_ hybrid plants (amphidiploids) under control and salt stress conditions. Bars represent means ± SE and bars with the same letter do not significantly differ at *p* < 0.05 among the genotypes.

ANOVA showed significant effects of salt stress, genotype, and salt stress × genotype interaction on the TPC and TFC. Salt stress caused an increase in TPC and TFC content in the leaves (Table 2), while much higher contents TPC content were observed in *Ae. cylindrica* genotype (21.7 mg TAE g^–1^ DW) and “Chinese Spring” × *Ae. cylindrica* amphidiploid genotypes (18.5 mg TAE g^–1^ DW) (Figure 2A). On the other hand, there was no significant difference in TPC among the three remaining genotypes (two bread wheat cultivars and “Roshan” × *Ae. cylindrica* amphidiploid) in salt stress conditions. TFC was likewise increased significantly by salt stress when compared with the control conditions. In contrast to the female parents (wheat cultivars), the male parent (*Ae. cylindrica*) displayed the highest TFC content (7.74 mg QE g^–1^ DW) under salt stress conditions. Although, “Chinese Spring” × *Ae. cylindrica* amphidiploid genotype showed responses intermediate between two parents however, no significant difference was found between “Roshan” × *Ae. cylindrica* amphidiploid genotype and its female parents (Figure 2B).

### DPPH Radical Scavenging Activity

Plants can withstand saline conditions by various mechanisms for reducing oxidative damage and maintaining redox homeostasis. DPPH free radical scavenging activity (DPPH-RSA) of the leaves was influenced significantly by salt stress, genotype, and salt stress × genotype interaction (Table 1). Scavenging activity on DPPH radical, expressed as IC50, in the five genotypes studied are given in Figure 2C. In control conditions, overall DPPH values ranged from 360 μg ml^–1^ in the most salt-tolerant genotype of *Ae. cylindrica* to 2890 μg ml^–1^ in “Chinese Spring” the most sensitive wheat cultivar. Likewise, a similar trend was evident for the scavaging of DPPH in salt stress conditions.

### Reducing Power

Reducing antioxidant power in the tested genotypes (wheat cultivars, *Ae. cylinrica* genotype and amphidiploids) was influenced significantly by salt stress, genotype, and salt stress × genotype interaction (Table 1). Figure 2D shows that the highest antioxidant activity occurred in *Ae. cylindrica* genotype containing the lowest IC50 (IC50 = 0.72 mg ml^–1^) under salt stress conditions, whereas it did not differ significantly from the amphidiploids. Moreover, wheat cultivar (“Chinese Spring” and “Roshan”) recorded the lowest activity under salt stress conditions. Although, IC50 value also exhibited similar trends for all genotypes in the control conditions, however, BHT was found to possess the most potent activity in both control and salt stress conditions.

### Polyphenol Components

**FIGURE 3:**
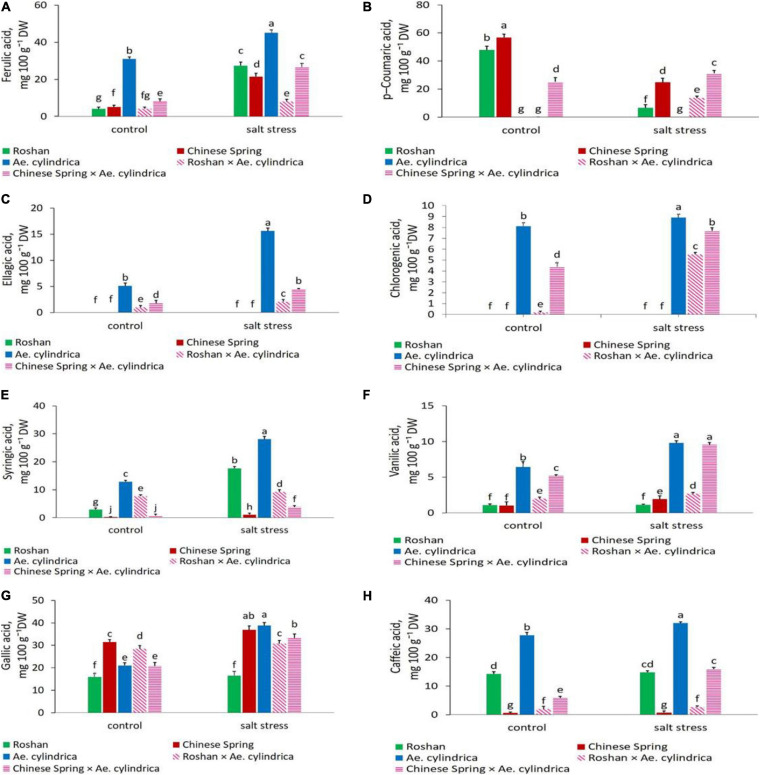
Mean comparison of **(A)** ferulic acid, **(B)** p-coumaric acid, **(C)** ellagic acid, **(D)** chlorogenic acid, **(E)** syringic acid, **(F)** vanillic acid, **(G)** gallic acid, and **(H)** caffeic acid of parents (*T. aestivum* “Roshan,” “Chinese spring,” *Ae. cylindrica*) and their F_1_ hybrid plants (amphidiploids) under control and salt stress conditions. Bars represent means ± SE and bars with the same letter do not significantly differ at *p* < 0.05 among the genotypes.

ANOVA results of the non-flavonoid polyphenol (phenolic acids) and flavonoid compounds detected by HPLC are summarized in Table 3. Significant changes in these compounds due to salt stress, genotype, and their interaction were observed. Table 4 shows the eight most abundant phenolic acids (chlorogenic acid, ellagic acid, ferulic acid, syringic acid, vanillic acid, caffeic acid, p-coumaric acid, and gallic acid) detected in the leaves. Table 4 also shows the three most abundant flavonoids, including flavones (luteolin and apigenin) and flavanols (rutin) identified in the genotypes. Salt stress induced both inhibitory and stimulatory responses in the synthesis of the phenolic acids. Salt stress increased all of the polyphenolic compounds including ellagic acid, ferulic acid, gallic acid, syringic acid, chlorogenic acid, vanillic acid, and caffeic acid, except for p-coumaric acid which was decreased (Table 4). In salt stress conditions, ferulic acid followed by gallic acid was the most abundant polyphenols (Table 4). The highest quantity of ferulic acid (45.08 mg 100 g^–1^ DW) was found in the leaves of the salt-tolerant, *Ae. cylindrica* genotype (Figure 3A). On the other hand, chlorogenic acid, ellagic acid, and vanillic acid showed the lowest quantity of all the compounds (Table 4).

**TABLE 3:** Analysis of variance of phenolic acids and flavonoids of the genotypes studied under control and salt stress conditions.

Source of variation	df	Chlorogenic acid	Ellagic acid	Ferulic acid	Syringic acid	Vanillic acid	p-Coumaric acid	Caffeic acid	Luteolin	Gallic acid	Apigenin	Rutin
Replication	2	0.5	0.12	3.41	0.85	0.14	55.82	10.73	0.01	372.1	8.35	1.98
Salt stress (S)	1	83.17**	63.24**	1596.3**	237.86**	10.89**	574.97**	8429.8**	70.22**	200664.7**	5183.1**	709.84**
Genotype (G)	4	388.07**	72.29**	662.19**	277.27**	191.58**	1122.67**	9324.5**	52.34**	60597.2**	1030.8**	597.60**
G × S	4	202.84**	18.98**	53.53**	35.18**	42.12**	594.95**	8525.2**	3.04**	83029.8**	924.99**	345.79**
Residual	18	0.18	0.03	0.21	0.09	0.12	87.83	5.87	0.76	86.36	1.65	0.30
CV (%)		6.48	5.24	2.76	3.71	7.07	45.49	8.14	10.28	4.15	3.81	4.10

**Significant at p < 0.01.

**TABLE 4:** Means (± SE) of phenolic acids and flavonoid contents of the leaves of the studied genotypes under control and salt stress conditions.

Content (mg 100 g^–1^ DW)	Control (0 mM NaCl)	Salt stress (250 mM NaCl)	LSD (*P* < 0.05)	Change (%)
Chlorogenic acid	4.24 ± 1.57	5.50 ± 0.40	0.43	29.72
Ellagic acid	1.37 ± 0.21	4.92 ± 0.60	0.17	259.12
Ferulic acid	7.82 ± 12.66	25.69 ± 1.26	0.48	228.52
Syringic acid	4.85 ± 0.51	11.75 ± 1.06	0.32	142.27
Vanillic acid	4.26 ± 0.90	5.73 ± 0.47	0.36	34.51
p-Coumaric acid	10.24 ± 3.20	6.96 ± 1.21	9.66	−32.03
Caffeic acid	9.25 ± 8.76	10.31 ± 1.65	2.50	11.46
Gallic acid	13.50 ± 1.13	23.82 ± 5.47	9.58	162.20
Luteolin	6.61 ± 0.22	10.36 ± 0.57	0.90	56.73
Apigenin	17.57 ± 1.33	20.76 ± 2.64	1.32	183.21
Rutin	7.36 ± 0.57	19.25 ± 1.97	0.56	161.55

Under salt stress conditions, p-coumaric acid content exhibited a decline relative to control in wheat cultivars while it was not affected in *Ae. cylinrica* and amphidiploids (Figure 3B). Interestingly, the amphidiploids exhibited an intermediate level of most of the detected polyphenols between the two parents.

**FIGURE 4:**
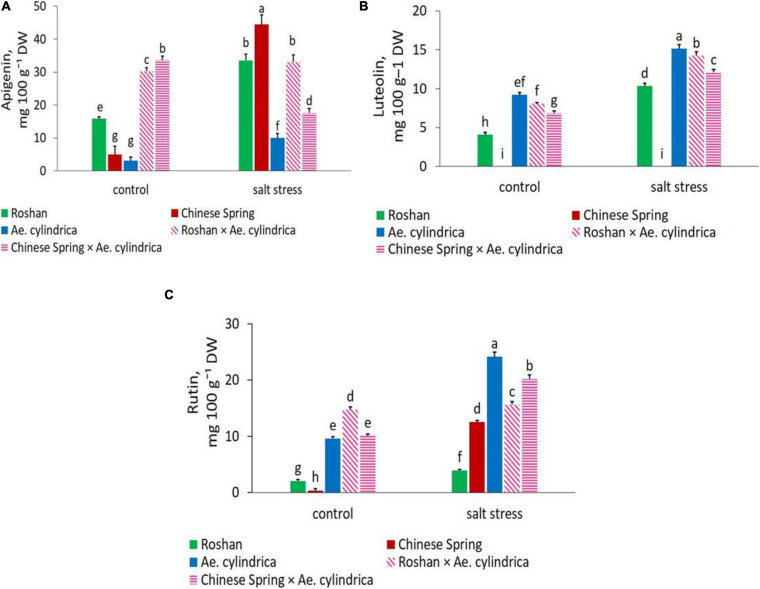
Mean comparison of **(A)** apigenin, **(B)** luteolin, and **(C)** rutin of parents (*T. aestivum* “Roshan,” “Chinese spring,” *Ae. cylindrica*) and their F_1_ hybrid plants (amphidiploids) under control and salt stress conditions. Bars represent means ± SE and bars with the same letter do not significantly differ at *p* < 0.05 among the genotypes.

Clearly, *Ae. cylindrica* showed significantly higher ellagic acid content (15.61 mg 100 g^–1^ DW) than the wheat cultivars under salt stress conditions (Figure 3C). Ellagic acid is a polyphenol compound with antioxidant properties and a derivative of gallic acid. Similar superiority of *Ae. cylindrica* was seen in chlorogenic acid (Figure 3D), vanillic acid (Figure 3F), gallic acid (Figure 3G), and rutin (Figure 4C) contents. On the other hand, in the salt stress conditions compared with the control ones, apigenin was significantly higher in “Chinese Spring” cultivar (16.9-fold) in salt stress conditions compared with control (Figure 4A), as well as the increased amount (3.2-fold) in *Ae. cylindrica* control conditions (data not shown).

The three flavonoid components were positively and significantly influenced by salt stress. In salt stress conditions, wheat cultivars (“Chinese spring” and “Roshan”) showed the highest content (44.48 and 33.56 mg 100 g^–1^ DW, respectively) of apigenin, while *Ae. cylindrica* had the lowest (10.01 mg 100 g^–1^ DW) (Figure 4A). In contrast, significantly higher amounts of the flavone (luteolin, 15.14 mg 100g^–1^ DW, Figure 4B) and flavanol (rutin, 24.12 mg g^–1^ DW, Figure 4C) were observed in *Ae. cylindrica* than the other genotypes.

### Relationships of Traits

A backward stepwise regression analysis was performed to examine which combination of variables best predicts yield loss due to salt stress. The results showed that proline followed by TPC and DPPH were the strongest predictors of yield loss under salt stress conditions (Table 5). Overall, this regression model could explain 91% of the variance in yield loss, and out of which, TPC (*r* = 0.76^∗∗^) contributed the most toward the variation in yield loss.

**TABLE 5:** Results of multiple regression analysis with yield loss as a dependent variable under salt stress conditions.

Independent variable^*a*^	Coefficient (B)	Standard error	Adjusted *R*^2*b*^ (R^2^)	*P*^*c*^
Intercept	143.78	20.64		0.0001
Proline	–1.18	0.6	0.41	0.0751
TPC	–2.95	0.51	0.58	0.0001
DPPH	–47.58	20.93	0.54	0.0441
*R*^2^ = 0.91 and Cp = 1.50				

*^a^Protein content, proline content, Electrolyte leakage (El) Total phenolic contents (TPC), Total flavonoid content (TFC), 2, 2-diphenyl-1-picrylhydrazyl (DPPH), and reducing power (IC50) as independent variables. ^b^Calculated by linear regression analysis. ^c^All variables left in the model are significant at the p < 0.1 level.*

Table 6 summarizes the correlation coefficients of the variables discussed below. TPC correlated negatively with DPPH (*r* = −0.66**). A negative correlation was found between TFC and DPPH scavenging rate (*r* = –0.58*) and also reducing power (*r* = −0.66^∗∗^). These results show that phenolic compounds (TPC and TFC) are positively associated with antioxidant activity. The correlation coefficients between the phenolic acids and the biochemical traits showed a positive between chlorogenic acid and DPPH scavenging rate (*r* = 0.58^∗^). Moreover, a positive correlation exists between ferulic acid content and TPC (*r* = 0.85^∗∗^).

**TABLE 6:** Correlation coefficients of biochemical traits, polyphenols and yield loss.

	Protein content	Proline content	EL	TPC	TFC	DPPH	Reducing power (RP)			Yield loss	
Protein	1										
Proline	0.56*	1									
EL	−0.70**	−0.77**	1								
TPC	0.53*	0.62*	−0.56*	1							
TFC	0.81**	0.55*	−0.60*	0.57*	1						
DPPH	0.10^*ns*^	−0.56*	−0.20^*ns*^	−0.66**	−0.58*	1					
RP	−0.60*	−0.86**	0.81**	−0.33^*ns*^	−0.66*	−0.23^*ns*^	1				
Yield loss	−0.56*	−0.55*	0.54*	−0.93**	−0.59*	0.65**	–0.21^*ns*^			1	

**Polyphenol**	**Protein**	**Proline**	**EL**	**TPC**	**TFC**	**DPPH**	**Reducing power**			**Yield loss**	

Chlorogenic	−0.05^*ns*^	0.61*	−0.26^*ns*^	0.63*	−0.06^*ns*^	0.78**	–0.22^*ns*^			–0.66**	
Ellagic	0.42^*ns*^	0.47^*ns*^	−0.42^*ns*^	0.92**	0.49^*ns*^	−0.58*	–0.16^*ns*^			–0.94**	
Ferulic	0.37^*ns*^	0.22^*ns*^	−0.36^*ns*^	0.85**	0.36^*ns*^	−0.36^*ns*^	0.02^*ns*^			–0.79**	
Syringic	0.49^*ns*^	0.44^*ns*^	−0.32^*ns*^	0.54*	0.74**	0.73**	–0.18^*ns*^			–0.85**	
Vanillic	−0.04^*ns*^	0.31^*ns*^	−0.23^*ns*^	0.62*	−0.19^*ns*^	−0.22^*ns*^	0.02^*ns*^			–0.65**	
p-Coumaric	−0.61*	0.44^*ns*^	0.38^*ns*^	−0.65**	−0.91**	0.64**	0.33^*ns*^			0.25^*ns*^	
Caffeic	−0.37^*ns*^	0.35^*ns*^	0.46^*ns*^	0.38^*ns*^	−0.21^*ns*^	0.70**	0.69**			–0.49^*ns*^	
Luteolin	−0.10^*ns*^	0.30^*ns*^	0.06^*ns*^	0.53*	0.17^*ns*^	0.91**	0.01^*ns*^			–0.64**	
Gallic	0.45^*ns*^	0.88**	−0.77**	0.62*	0.26^*ns*^	0.08^*ns*^	–0.73**			–0.49^*ns*^	
Apigenin	−0.11^*ns*^	−0.41^*ns*^	0.20^*ns*^	−0.37^*ns*^	−0.23^*ns*^	0.75**	–0.01^*ns*^			0.42^*ns*^	
Rutin	0.39^*ns*^	0.75**	−0.56*	0.94**	0.41^*ns*^	−0.50^*ns*^	–0.41^*ns*^			–0.89**	

**Polyphenol**	**Ferulic acid**	**Syringic acid**	**Vanillic acid**	**p-coumaric acid**	**Caffeic acid**	**Luteolin**	**Gallic acid**	**Apigenin**	**Rutin**	**Chlorogenic**	**Ellagic**

Ferulic	1										
Syringic	0.69**	1									
Vanillic	0.71**	0.27^*ns*^	1								
p-Coumaric	−0.45^*ns*^	−0.93**	0.08^*ns*^	1							
Caffeic	0.59*	0.44^*ns*^	0.53*	−0.15^*ns*^	1						
Luteolin	0.21^*ns*^	0.64**	0.36^*ns*^	−0.48^*ns*^	0.50^*ns*^	1					
Gallic	0.33^*ns*^	0.23^*ns*^	0.60*	−0.12^*ns*^	−0.27^*ns*^	0.24^*ns*^	1				
Apigenin	0.77**	−0.77**	−0.78**	0.48^*ns*^	−0.70**	0.76**	−0.44^*ns*^	1			
Rutin	0.71**	0.72**	0.76**	−0.50^*ns*^	0.31^*ns*^	0.64**	0.78**	−0.89**	1		
Chlorogenic	0.37^*ns*^	0.37^*ns*^	0.79**	−0.10^*ns*^	0.34^*ns*^	0.75**	0.72**	−0.79**	0.83**	1	
Ellagic	0.92**	0.85**	0.71**	−0.63*	0.55*	0.53*	0.49^*ns*^	−0.92**	0.90**	0.59*	1

Protein content (Protein), Proline content (Proline), Electrolyte leakage (EL), Total phenolic contents (TPC), Total flavonoid content (TFC), and 2, 2-diphenyl-1-picrylhydrazyl (DPPH). ^ns^ not significant. * and ** significant at p < 0.05 and p < 0.01, respectively.

The results of correlation coefficients between yield loss and other traits are in Table 6. Yield loss was positively and significantly correlated with EL (*r* = 0.54^∗^) and DPPH (*r* = 0.65^∗∗^); while negatively correlated with protein content (*r* = –0.56^∗^), proline content (*r* = –0.55^∗^), TPC (*r* = –0.93^∗∗^), and TFC (*r* = –0.59^∗^). These relationships were consistent with observations in salt-tolerant genotypes having higher protein content, proline content, TPC, and TFC as well as, lower EL and yield loss (Figures 1, 2). Strong positive correlation was detected between DPPH and chlorogenic (*r* = 0.78^∗∗^), luteolin (*r* = 0.91^∗∗^), caffeic acid (*r* = 0.70^∗∗^), and apigenin (*r* = 0.75^∗∗^). A positive correlation was also found between luteolin and chlorogenic acid (*r* = 0.75^∗∗^); apigenin and ferulic acid (*r* = 0.77^∗∗^); as well as luteolin and apigenin (*r* = 0.76^∗∗^).

## Discussion

The leaf proteins are vastly critical in the growth, reproduction, and ultimate grain yield of the plants. The reduction of protein content in the plant leaves due to salt stress is not surprising since it is well established that the initial targets of ROS are proteins in biological systems. The chloroplast is one of the primary targets of ROS, which causes marked alterations in a wide variety of proteins such as thylakoid and stromal, including degradation and inactivation of Rubisco (Mehta et al., 1992; Ishida et al., 1997). Therefore, leaf protein content is one of the crucial indicators of the effects of salt stress (Isayenkov and Maathuis, 2019). Genetic variation for plant leaf protein affected by salt stress is one of the contentious areas in the literature. The relationship of genetic background and the effects of salt stress on protein content has not extensively been explored in the literature. Moreover, the effect direction of salinity is a consensual issue, which differs widely in the literature. For instance, in wheat, Radi et al. (2013) reported that total protein content in the shoots of two cultivars (tolerant and sensitive) was decreased analogously by salt stress. On the other hand, leaf protein content that differentiated genotypically was found to be increasing with salt stress (Afzal et al., 2006).

Increased accumulation of compatible solutes like proline has been suggested to enhance salt tolerance. Eventually, proline, likewise other osmolytes, modulate redox potential by conferring osmotic adjustment, protecting cellular membranes, and stabilizing enzymes under abiotic stress (Romero-Aranda et al., 2006). Arabbeigi et al. (2018) suggested that higher expression of the gene responsible for proline biosynthesis (*P5CS*) in *Ae. cylindrica* may correlate with salt tolerance. This finding is in agreement with Kumar et al. (2017) in wheat. This is partially consistent with observations on barley by Ebrahim et al. (2020), who find that salt stress caused a massive accumulation of proline, but noted a higher accumulation of proline in the leaves of salt-sensitive genotypes than salt-tolerant ones. Therefore, the osmoadaptive response involves the accumulation of proline, which underline an unspecific role in the tolerance to abiotic stress (such as drought and salinity) is a matter of debate. There are a number of possible reasons for this inconsistency. The most prominent among them are genotype/species, stress intensity, stress duration, and physiological stage differences between studies (Ebrahim et al., 2020).

In plant cells, the preservation of plasmid membrane integrity is a critical adaptive strategy against free radicals (Kaya et al., 2009; Isayenkov and Maathuis, 2019). In the current study, higher electrolyte leakage was found in the female parents (wheat cultivars) than the hyper-salt tolerant *Ae. cylindrica* and amphidiploid genotypes under stress conditions.

These findings support the idea that plasma membrane may represent a promising strategy for improving the efficiency in regulating transmembrane ion and metabolite fluxes during the stress. The results of the current study are consistent with those of Radi et al. (2013), who observed an increase in EL in the wheat genotypes due to salt stress and also found that the salt-sensitive genotype had higher EL values than the salt-tolerant one.

There are many biochemical mechanisms that protect plants against the harmful influences of salt stress. Phenolic compounds are not only the most abundant secondary metabolites in the plant kingdom but also are the most crucial antioxidants for scavenging the excessive ROS that is generated by the majority of stressors. Flavonoids, as a group belong to phenolic compounds, are also known to have antioxidant properties (Tohidi et al., 2017). Our result showed a significant increase in total phenolic and flavonoids compounds in response to salt stress. This agrees with the observations made by Hichem et al. (2009), who reported the significant effect of salt stress on the total phenolic and flavonoids compounds in two maize (*Zea mays* L.) cultivars.

Over the past several decades, researchers have consistently found that the strong association between polyphenols and abiotic-stress tolerance is an excellent predictive of the extent of patience, and hence can be used as an indicator of maintenance of the redox state in the cells (Hodaei et al., 2018; Sharma et al., 2019). While there are enormous bodies of literature that specifically address phenolic compounds of the edible plant parts (e.g., seeds in cereals), mainly because of the interest in health benefits of polyphenol consumption, paucity remains on research that investigates the motivations behind the ameliorating effect of polyphenols on the phytotoxicity of photosynthetic tissues caused by salt stress. Martinez et al. (2016) reported an increase in accumulation of flavonoids in tomato plants in response to abiotic stress coincides with dual protective effect as antioxidant against oxidative damage induced by the stress, and subsequently as the health-promoting compounds of edible plants.

The results of the current study clearly show that wheat and Aegilops genotypes differ for the accumulation of the polyphenols (TPC and TFC) in their leaves. These compounds were significantly increased in response to salt stress. Kumar et al. (2017) have also reported that salt stress-triggered a significant increase in TPC in salt-tolerant wheat genotype. A significant genotypic difference was observed on the TPC in durum wheat grains (Boukid et al., 2019). The result of the current study generally indicated that *Ae. cylindrica* (male parent) had higher antioxidant activity than female parents (wheat cultivars) in both control and salt stress conditions. Further, the results also show that in comparison with female parent, amphidiploid plants had higher antioxidant activities expressed by DPPH (IC50). Specifically, the research presented here is not only motivated but also supported our initial works bestowing genotypic differences for antioxidant enzymes, malondialdehyde (MDA), and H_2_O_2_ in the same set of genotypes (Kiani et al., 2021).

The reducing power is another protective mechanism of oxidative stress, which reveals the electron-donating ability of the natural extracts and cellular redox homeostasis. Non-enzymatic antioxidants can be defined as inactivation of oxidants by reductants in redox reactions in which one reactive species is reduced at the expense of the oxidation of another (Benzie and Strain, 1996; Wang et al., 2014). The assessment of reducing power is used to investigate the complex impression of natural extracts and to explore the redox homeostasis in leaf tissue homogenates. In the current study, the ferric reducing antioxidant power of the samples were compared with a synthetic antioxidant BHT (IC50 = 0.36 mg ml^–1^). The plant samples with stronger reducing powers are capable of donating their electrons to the ROS and induce degeneration. In this way, *Ae. cylindrica* and two amphidiploids were a superior group of genotypes with the lowest IC50 values.

In the current study, chlorogenic acid, caffeic acid, ellagic acid, ferulic acid, gallic acid, p-coumaric acid, syringic acid, and vanillic acid were the most abundant phenolic acids detected. The accumulation of these phenolic acids was increased in the leaf tissues by salt stress except for p-coumaric that decreased. Our results also revealed that three most abundant flavonoids, including flavones (luteolin and apigenin) and flavanols (rutin) identified in the genotypes. Increased accumulation of phenolic acids in the leaves of *Amaranthus tricolor* L. (a leafy vegetable) in response to salt stress has already been found, including caffeic acid, chlorogenic acid, ferulic acid, gallic acid, 4-hydroxybenzoic acid, p-coumaric acid, salicylic acid, sinapic acid, and vanillic acid (Sarker and Oba, 2018). Interestingly, amphidiploid genotypes showed an intermediate level between the two parents for most of the detected polyphenols, and provides support for our hypothesis and clues to their contribution toward diminishing yield loss under saline conditions. These data, together with the differential accumulation of TPC and TFC, suggest further evidence that phenolic compounds play vital physiological and biochemical roles in plant cells, particularly helping ameliorate abiotic stress (Sharma et al., 2019). Moreover, our observation of ferulic acid as the major phenolic compound in the leaves, provides supportive evidence for the existence of a similar result for the wheat grains (Adom and Liu, 2002; Boz, 2015; Ma et al., 2016). Ma et al. (2016) observed that ferulic acid accounts for 87.10–90.60% of the TPC, ranging from 21.88 to 37.31μg g^–1^ in the white and purple grains of wheat, respectively.

Grain yield is a key to understand the link between biochemical responses in plant and anthropogenically imposed stressor, on one hand, and how this factor influences the adaptation through genetic diversity on the other (Arzani and Ashraf, 2016, 2017). Variation in grain yield depends on both diverse genetic of wheat genotypes and differential response to prevalent environmental conditions at the grain filling stage. Salt stress negatively affects grain yield as the outcome of the physiological and biochemical changes. A negative effect of salt stress on grain yield has also been shown by Chamekh et al. (2016), who observed that the salinity of the irrigation water at 16 dS m^–1^ mM NaCl significantly decreased grain yield. This study support the hypothesis that there is a trade-off between reproduction and stress tolerance (Araus et al., 2008).

In addition, negative and strong correlation coefficients of TPC and TFC with IC50 of DPPH radical indicated that the phenolic compounds contribute to antioxidant potential in the studied genotypes. Likewise, a positive correlation has been reported between RSA and TPC in cereals (Dykes and Rooney, 2007). We also found that the hyper salt-tolerant genotype (*Ae. cylindrica*) had the most substantial inhibition on the DPPH radical among the genotypes studied. These findings are partially consistent with those of Kumar et al. (2017), who found genotypic variation and significant increment in scavenging of DPPH (%) in the seedling shoots of two out of four wheat cultivars in response to salt stress, on the one hand, and confirmed the antioxidant activity of TPC on the other. In addition, a positive correlation was established between ferulic acid content and TPC. This finding is consistent with the significant relationship that exists between ferulic acid content and TPC in triticale genotypes under drought stress conditions (Hura et al., 2007). In the current study, chlorogenic acid content was correlated significantly with DPPH scavenging rate. This is in accordance with the work of Yan et al. (2016), based on herb plant (*Origanum vulgare*), which showed that increase in chlorogenic acid correlates significantly with the DPPH scavenging responses evoked by water stress.

Ferulic acid is a hydroxycinnamic acid and a precursor of lignin and vanillic acid biosynthesis with good antioxidant and anti-aging functions. It involves not only an inhibitor of the enzyme catalyzing the formation of free radical species and an enhancer of scavenger enzyme activity but a free radical scavenger. Therefore, salt stress might activate lignification inhibitors leading to accumulation of ferulic acid (Boz, 2015). Association does not always imply causation, however, and there is still no overwhelming evidence that the role of these phenolic acids being recognized as a plausible strategy to help alleviate the stress in wild plant species. The positive correlations between luteolin and chlorogenic acid, apigenin and ferulic acid, as well as luteolin and apigenin may be explained by shared biosynthetic pathways. Consistent with our results is the observation that a positive association between the antioxidant activity and luteolin content has been described (Wang et al., 2014). Based on multivariate regression analysis, RAS, TFC, and DPPH were significantly contributed to the salt tolerance. Phenolic and flavonoid compounds are an essential non-enzymatic antioxidant involved in the scavenging of ROS (Tohidi et al., 2017; Sharma et al., 2019), and in particular those evoked by oxidative stress during salt stress (Chen et al., 2019). The strong relationships between yield loss and biochemical traits coincide with higher protein content, proline content, TPC, and TFC, as well as, lower EL and yield loss in salt-tolerant genotypes. On the whole the data yield the conclusion that (1) these biochemical compounds play a crucial role in protecting plants against damage by salt stress and (2) these variables would be useful for the prediction of yield-loss caused by salt stress in Triticeae tribe.

## Conclusion

To our knowledge, this is the first report that establishes the antioxidant and protective role of polyphenols against salt stress-induced oxidative stress and the differential response of genotypes, using highly salt-tolerant (*Ae. cylindrica*), moderately salt-tolerant (amphidiploids), and salt-sensitive (*T. aestivum*) genotypes. The vigorous antioxidant activity and robust accumulation of phenolic compounds in the leaves of the male parent (*Ae. cylindrica* Host) and amphidiploid derivates would imply greater sophistication in genetic diversity for the evolvement of defense-oriented strategies to prevent the accumulation of intracellular free-radicals generated under salt stress.

## Data Availability Statement

The raw data supporting the conclusions of this article will be made available by the authors, without undue reservation.

## Author Contributions

AA conceived and designed the research. RK conducted the experiments under the supervision of AA and SAMMM. RK performed the overall data analysis, and also wrote down the draft of the manuscript with significant inputs from AA and SAMMM. All authors contributed to the article and approved the submitted version.

## Conflict of Interest

The authors declare that the research was conducted in the absence of any commercial or financial relationships that could be construed as a potential conflict of interest.
